# Artificial Intelligence in Pediatric Cardiology: Present Applications and Future Directions

**DOI:** 10.3390/pediatric18030070

**Published:** 2026-05-25

**Authors:** Bianca Ada Magnanini, Irene Raso, Sara Santacesaria, Gaia Dell’Acqua, Savina Mannarino

**Affiliations:** 1 Centro Cardiologico Monzino IRCCS, Department of Clinical Sciences and Community Health, University of Milan, 20122 Milan, Italy; 2 Pediatric Cardiology Unit, Buzzi Children’s Hospital, 20154 Milan, Italy; santacesaria.sara@asst-fbf-sacco.it (S.S.); savina.mannarino@asst-fbf-sacco.it (S.M.); 3 Pediatric Department, Buzzi Children’s Hospital, University of Milan, 20154 Milan, Italy; gaia.dellacqua@unimi.it

**Keywords:** artificial intelligence, pediatric cardiology, pediatric cardiac surgery, machine learning, deep learning, congenital heart disease

## Abstract

Artificial intelligence (AI) is rapidly transforming cardiovascular medicine, with growing applications in pediatric cardiology. AI techniques, particularly machine learning and deep learning, enable the analysis of complex and heterogeneous data, supporting diagnosis, risk stratification, and clinical decision-making. This paper provides an overview of current AI applications in this field, discusses existing challenges, and explores future perspectives. In pediatric cardiology, AI has shown promising results across multiple domains. In electrocardiography, AI algorithms improve diagnostic accuracy and enable early detection of cardiac conditions, even in asymptomatic patients, while facilitating telecardiology-based care pathways. In cardiac auscultation, AI-assisted digital stethoscopes enhance the distinction between innocent and pathological murmurs, supporting primary care physicians and optimizing referral to pediatric cardiologic centers. Multimodality imaging represents one of the most advanced areas of AI applications. In echocardiography, magnetic resonance and computed tomography, AI improves image acquisition, view classification, and automated quantification, contributing to more standardized and reproducible assessments. Additionally, emerging technologies such as virtual reality, integrated with AI, offer innovative tools for education, surgical planning, and patient-specific modelling. Despite these advances, several limitations remain, including limited availability of large pediatric datasets, challenges in model generalizability and issues related to interpretability and integration into clinical workflows. In conclusion, AI represents a powerful complementary tool in pediatric cardiology, with the potential to improve diagnostic accuracy, optimize healthcare resources and support the transition toward precision medicine.

## 1. Introduction

Artificial intelligence (AI) is revolutionizing healthcare by offering innovative solutions for patient diagnosis, treatment and management. In the current medical landscape, AI integration is no longer confined to theoretical research: the use of machine learning (ML) and deep learning (DL), the main applications of AI, has already been studied and implemented in adult cardiology [1,2]. Only recently has AI begun to expand into the field of pediatric cardiology. In this context, the potential of AI is particularly relevant, especially in the field of congenital heart disease (CHD) [3], a highly complex area requiring multiple diagnostic and therapeutic approaches.

AI applications in this domain are numerous and range from pre- and post-natal screening strategies (fetal echocardiography, cardiac auscultation, pulse oximetry and electrocardiography) to multimodal imaging (echocardiography, cardiac magnetic resonance and computed tomography). Furthermore, AI is increasingly applied to procedural planning, risk stratification, and outcome prediction, as well as to the development of precision medicine approaches [4].

The purpose of this document is to describe the current state of the art of AI applications in pediatric cardiology, to analyze the main existing challenges and explore future opportunities and perspectives.

The added value of this paper lies in its broad, integrated and clinically oriented approach: rather than focusing on a single AI application or imaging modality, this manuscript provides an integrated overview of AI across the entire spectrum of sectors in pediatric cardiology—from early screening tools to advanced cardiac imaging and immersive technologies, with particular application for procedural planning, risk stratification and future precision medicine applications. Another important aspect is its translational and clinical perspective: AI applications are discussed both from a technical standpoint and in relation to their potential impact on clinical practice, diagnostic-therapeutic management and patient-centered care. Particular emphasis is placed on congenital heart disease, a uniquely complex field in which anatomical heterogeneity, age-related physiological changes, limited datasets, and the need for multimodality assessment create both major challenges and important opportunities for AI-based innovation, including insight into ethical and regulatory aspects. Overall, this paper aims to provide clinicians with a structured overview of the current state of the art and the major barriers to clinical implementation.

## 2. Methods

This article is a narrative review of applications of artificial intelligence in pediatric cardiology. A structured literature search was performed using the electronic database PubMed and was last updated in February 2026. No time restrictions were applied. The following keywords and their combinations were used: “artificial intelligence”, “machine learning”, “deep learning”, “pediatric cardiology”, “congenital heart disease”, “electrocardiogram”, “cardiac auscultation”, “echocardiography”, “cardiac magnetic resonance”, “cardiac computed tomography” and “virtual reality”. The search was limited to English-language papers and considered original articles, case reports, meta-analyses, reviews and systematic reviews. Papers were selected according to their relevance to the main topics of the review, with priority given to studies involving pediatric populations, congenital heart disease, multimodality imaging, diagnostic support, workflow optimization, risk stratification, and emerging technologies. The list was also manually screened in order not to miss additional studies of interest. Adult cardiology studies were included when deemed relevant. This review was not designed as a systematic review or meta-analysis; therefore, no formal quality assessment or quantitative synthesis was performed.

## 3. Brief Overview of Artificial Intelligence

**Figure 1:**
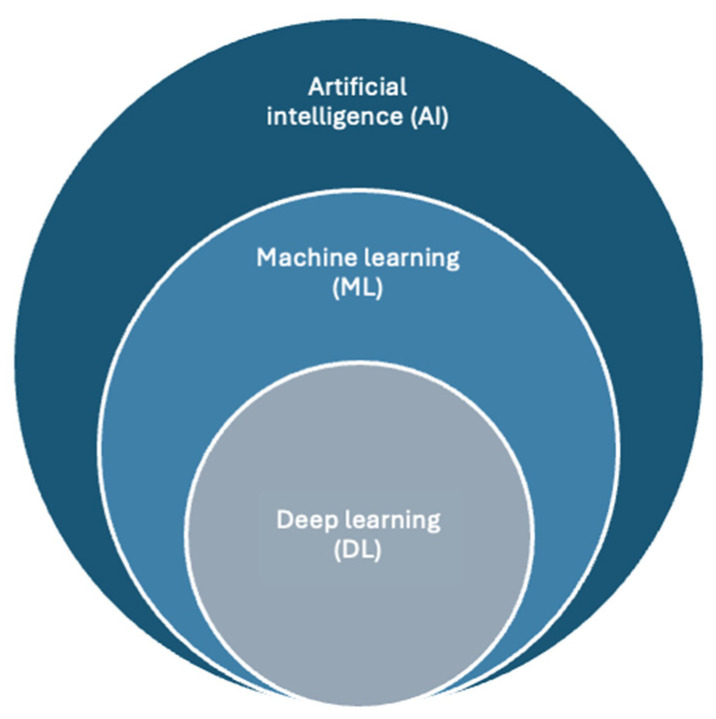
Flowchart of AI techniques.

Artificial intelligence (AI) refers to computational systems designed to automatically perform intellectual tasks that typically require human intelligence, such as pattern recognition, decision-making and data interpretation. In medicine, the main approaches enabling these capabilities are machine learning (ML) and deep learning (DL) [5] [Figure 1].

Machine learning is a set of AI based on algorithms that learn from data to identify patterns and make predictions without being explicitly programmed [6]. ML models can be broadly classified into four main categories: (i) supervised learning, (ii) unsupervised learning, (iii) semi-supervised learning, and (iv) reinforcement learning. Supervised learning uses labeled data to learn the relationship between input features and a target outcome, allowing the model to make predictions on new data (task-driven approach, i.e., regression and classification). In contrast, unsupervised learning analyzes unlabeled data to identify underlying patterns or structures, grouping similar instances into categories without predefined labels (data-driven approach, i.e., clustering and association). Semi-supervised learning can be defined as a hybrid method that combines labeled and unlabeled data. A small set of labeled examples guides the model, while larger amounts of unlabeled data are used to improve learning. This approach is particularly useful when labeled data are limited. Reinforcement learning is a system that learns optimal actions through interactions with an environment, guided by rewards and penalties. It is mainly suited for complex, dynamic tasks rather than simple problems, and its use in medicine is currently limited and beyond the scope of this review.

Deep learning is a set of machine learning techniques that relies on multi-layered neural networks capable of progressively extracting higher-level features from raw data [7,8,9]. The main advantage of deep learning over traditional machine learning methods is its superior performance in many scenarios, particularly when handling large datasets. It has demonstrated strong performance in a wide range of domains, including image analysis, natural language processing, speech recognition and genomics [10]. The most common DL algorithms are Multi-Layer Perceptron (MLP), convolutional neural network (CNN) and recurrent neural network (RNN). A multilayer perceptron is a basic deep learning architecture composed of fully connected layers (input, hidden, and output) that learn through backpropagation. Convolutional neural networks extend this model by exploiting spatial structure, making them particularly effective for image and video analysis. Recurrent neural networks, including long short-term memory (LSTM) networks, are designed to handle sequential data by capturing temporal dependencies, and are widely used for time-series and language tasks (i.e., electrocardiographic signals) [11].

In clinical practice, AI systems are trained on large datasets to recognize patterns associated with specific diseases, enabling applications such as diagnosis, risk stratification and outcome prediction [12]. However, their performance depends on the quality and representativeness of the training data, and their integration into clinical workflows requires careful validation, algorithm transparency, interpretability and continuous monitoring of accuracy and performance [13].

## 4. Cardiac Auscultation

Artificial intelligence-based digital support is emerging as a valuable tool for the interpretation of cardiac sounds. One of the main applications in this field involves AI-assisted cardiac auscultation through the use of electronic stethoscopes. In addition, integration with Bluetooth-enabled smartphone applications allows for the recording of heart sounds at different auscultatory sites. These devices can capture acoustic signals and convert them into electrical signals that can be amplified, stored, replayed and transmitted for remote specialist evaluation, making them particularly suitable for telemedicine applications [14].

Cardiac auscultation in pediatric patients—particularly the distinction between physiological and pathological murmurs—remains a challenging task. Several studies have demonstrated suboptimal accuracy not only among pediatricians but also among cardiology trainees and general practitioners. This limitation may lead to unnecessary referrals or, conversely, missed diagnoses [15,16].

AI algorithms can enhance auscultation by filtering background noise and amplifying clinically relevant heart sounds, thereby improving diagnostic accuracy and facilitating early detection of cardiac abnormalities. Recent evidence highlights the transformative potential of AI-assisted digital auscultation devices in pediatric care, especially when integrated with telemedicine systems [17,18].

A key advantage of this technology is its applicability in community-based pediatric care. By supporting pediatricians in the initial evaluation of heart murmurs, AI-assisted auscultation may reduce unnecessary referrals to tertiary centers and optimize healthcare resources, including waiting lists. At the same time, it may serve as a complementary tool for specialists in the diagnosis and follow-up of congenital heart disease, an area in which accuracy and clinical applicability are of increasing interest [19].

At our Pediatric Cardiology Center at Vittore Buzzi Children’s Hospital, an AI-based deep learning algorithm is currently under development in collaboration with a team of engineers. The system is designed to recognize and classify heart sounds (data not yet published).

## 5. Electrocardiogram

The application of AI to the electrocardiogram (ECG) represents a rapidly evolving field.

The ECG contains a large amount of physiological information that can be used to identify and characterize a wide range of health conditions. AI has the potential to transform this non-invasive and widely available examination into a powerful tool for screening and prediction of both cardiac and non-cardiac diseases, even in asymptomatic individuals.

In adult populations, supervised AI algorithms have demonstrated the ability to detect and predict conditions such as paroxysmal atrial fibrillation from ECGs recorded during sinus rhythm, left ventricular systolic dysfunction, channelopathies—even when electrocardiographically concealed—valvular heart disease, and hypertrophic cardiomyopathy [20].

Pediatric cardiovascular diseases differ significantly in etiology compared to adult acquired heart disease. They are mainly represented by congenital heart defects, along with arrhythmias and cardiomyopathies, while ischemic heart disease is rare. The management of pediatric patients with suspected cardiac disease often relies on primary care physicians, who play a key role in early screening through clinical examination and growth monitoring, including the detection of heart murmurs and ECG abnormalities. However, ECG interpretation in children remains challenging due to age-related physiological variations and the heterogeneity of congenital heart diseases, which may produce a wide spectrum of electrocardiographic patterns (including minimal or absent abnormalities even in complex conditions).

In Italy, an additional challenge is represented by the widespread use of ECG screening for competitive sports eligibility from early childhood, often performed in settings with limited expertise in pediatric cardiology. This highlights the need to make pediatric ECG interpretation more accessible, standardized and reliable, similar to what has been achieved in adult populations.

The main architectures used include convolutional neural networks (CNNs) and recurrent neural networks (RNNs), which enable the analysis of large volumes of electrocardiographic data and the identification of features not immediately recognizable through traditional clinical interpretation.

Several studies have demonstrated that deep learning models can accurately identify pathological patterns in pediatric populations, including hypertrophic cardiomyopathy, atrial septal defects and congenital long QT syndrome [21,22,23,24].

Furthermore, the integration of AI into telecardiology systems represents a practical and scalable solution. The ability to transmit ECG recordings from peripheral centers to specialized hospitals, combined with automated analysis, allows for early patient stratification and more appropriate referrals [25].

Despite these promising results, several challenges remain. These include the limited interpretability of AI models and the need for large, high-quality and representative datasets for training—key factors for broader adoption in clinical practice. In addition, the variability of ECG manifestations in congenital heart disease requires highly sensitive and robust algorithms.

Overall, the application of AI to pediatric ECG has the potential to improve healthcare efficiency by supporting clinicians in data interpretation, accelerating the diagnostic process, particularly in complex cases, and reducing unnecessary referrals to specialized centers.

## 6. Cardiovascular Multimodality Imaging

Pediatric cardiac imaging represents a particularly challenging area for the application of artificial intelligence, as cardiac structures are small, heart rates are high, and the clinical questions to be addressed are complex. The current implementation of AI in this field is mainly driven by machine learning (ML) and deep learning (DL) techniques, which are increasingly applied to echocardiography, cardiac magnetic resonance (CMR), and cardiac computed tomography (CCT).

### 6.1. Echocardiography

Echocardiography represents a crucial tool for diagnosis and longitudinal follow-up in pediatric cardiology. However, the application of AI in this field is considerably more challenging than in adult cardiology because of the marked anatomical and physiological heterogeneity of pediatric patients. In children with CHD, cardiac morphology may vary widely even among patients sharing the same diagnosis, while age-related physiological changes and growth-related modifications further increase variability, also in structurally normal hearts. In addition, pediatric echocardiography remains highly operator dependent. Image quality, acquisition of standard views and quantitative measurements are strongly influenced by probe positioning, insonation angle, patient cooperation, and the appropriate selection of ultrasound probes with different frequencies and frame rates.

**Figure 2:**
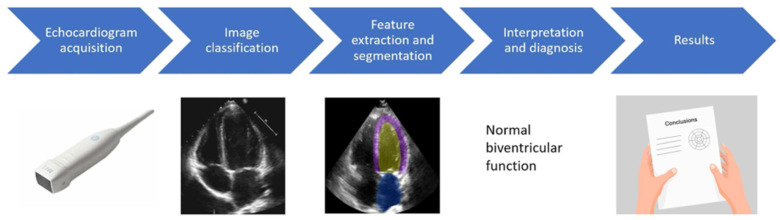
Flowchart for use of AI in echocardiography.

Even with the challenges it faces, AI has already demonstrated substantial value in adult echocardiography, where AI has the potential to support the entire workflow—image acquisition, quality optimization, view classification, automated segmentation, quantitative analysis, interpretation, disease detection, and prognostic stratification. Recently, these technologies have progressively expanded into pediatric echocardiography [26] [Figure 2].

A crucial prerequisite for accurate AI-based diagnosis of CHD is view classification. Correct identification of standard echocardiographic views ensures that all essential diagnostic projections are acquired and analyzed, reducing errors related to incomplete or suboptimal examinations, and represents the foundation for automated quantitative analysis and diagnosis.

Gearhart et al. [27] developed a convolutional neural network model trained on more than 12,000 pediatric echocardiographic images: the model was capable of autonomously classifying 27 standard pediatric echocardiographic views, including anatomical sweeps, color Doppler and Doppler tracings. The model achieved approximately 90% overall accuracy across all pediatric age groups, establishing an important foundation for future AI-assisted pediatric echocardiographic workflows. 

More advanced models enable automated segmentation of cardiac chambers and calculation of functional parameters, such as ejection fraction and cardiac output, with performance comparable to that of expert operators. Guo et al. [28] proposed a dual-attention enhancement feature fusion network for segmentation and quantitative analysis of pediatric echocardiography. The model was based on a fusion architecture, which combined channel attention mechanisms—to emphasize the most informative features, with spatial attention mechanisms—that help select the most relevant spatial information within the image—and with hybrid loss functions—designed to address pixel-level misalignment and boundary ambiguities. This approach enabled automated segmentation, followed by subsequent extractions of clinically relevant parameters, including ventricular and atrial dimensions and chamber areas. The model was applied to approximately 4500 pediatric echocardiographic images and achieved superior segmentation performance compared with other state-of-the-art approaches. Additionally, Lukyanenko et al. [29] developed EchoFocus-Measure, a large-scale AI platform designed to automatically extract both quantitative and qualitative echocardiographic parameters from complete pediatric echocardiographic studies. Using transformer-based architectures trained on millions of echocardiographic images and videos, the model demonstrated robust performance across children of different ages and with complex congenital heart disease, particularly for the quantitative assessment of left ventricular ejection fraction. These findings suggest the possible use of AI for a large-scale and global cohort of patients, expanding access to high-quality pediatric care.

AI applications have also expanded into automated detection of congenital heart defects. Jiang et al. [30] developed a deep learning model for automated detection of CHD, based on the analysis of seven echocardiographic views (including grayscale, color Doppler and bimodal images). The rationale behind this seven-view approach was that providing the model with multiple views would allow a broader analysis and improve the ability to recognize abnormalities. The images were used as input for a CNN trained to distinguish normal children from those with CHD. The model was tested on more than 14,000 images and achieved high diagnostic performance when appropriate image modalities were used. Erno et al. [31] proposed a convolutional neural network to identify patent ductus arteriosus (PDA) in premature infants using echocardiographic clips. More than 2000 videos were reviewed by a cardiologist and labeled as “PDA present/absent/indeterminate,” and the model demonstrated strong performance in classifying clips with and without PDA. Additional studies have developed models for the detection of other common CHDs (such as atrial septal defects) and Kawasaki disease [32,33,34].

Bridging these domains, Diller et al. [35] provided an example of an AI approach that combines both diagnostic classification and anatomical segmentation in complex CHD. In their study, a deep learning algorithm was applied to echocardiograms of patients with a systemic right ventricle (including patients with transposition of the great arteries after atrial switch repair and congenitally corrected transposition of the great arteries). CNNs were trained to classify the images into normal anatomy, atrial-switch TGA or ccTGA. In parallel, a U-Net architecture was trained on expert-annotated frames to automatically identify and segment the systemic ventricle. By combining apical and short-axis views, the model achieved high diagnostic accuracy, and automated ventricular segmentation showed agreement comparable to human experts. 

Interestingly, AI techniques are increasingly being applied to disease characterization, aiming to identify distinct phenotypic subgroups within apparently homogeneous patient populations. This approach is particularly innovative because it may reveal subtle, data-driven patterns that are not readily detectable by human assessment, but may correspond to different clinical trajectories, therapeutic needs and outcomes. Within this framework, Meza et al. [36] applied unsupervised cluster analysis to a large multicenter cohort of neonates with critical left heart obstruction. Echocardiographic studies were analyzed by a single cardiologist, who described more than 130 qualitative and quantitative variables related to left-sided cardiac structures; these data were then used as input for the cluster analysis. The model identified three distinct patient clusters, primarily driven by differences in left ventricular size and aortic valve morphology, associated with different management strategies and mortality rates. These results suggest that AI analytics may support disease classification beyond traditional anatomical categorizations.

Ultimately, AI-based echocardiographic analysis is also being explored as a tool for disease evolution prediction. Sun et al. [37] used natural language processing to extract structured variables from echocardiographic reports and medical records of children with perimembranous ventricular septal defect, developing a machine learning model that accurately predicted spontaneous closure at 1, 3, and 5 years. Similarly, Sharma et al. [38] developed a multimodal CNN combining pretreatment echocardiographic clips and perinatal data to predict pharmacologic closure of patent ductus arteriosus in premature infants. These studies suggest that AI analytics may expand the role of echocardiography toward individualized prediction of disease trajectory and treatment response.

Taken together, these approaches represent an important step toward the development of advanced decision-support systems. By improving standardization, quantitative analysis and reproducibility, AI may help clinicians integrate complex imaging data into more individualized diagnostic, prognostic and therapeutic pathways, while remaining complementary to expert clinical judgment.

### 6.2. Cardiac Magnetic Resonance

Pediatric cardiovascular magnetic resonance (CMR) is a highly informative modality. However, CMR acquisition and interpretation in children remain challenging, because of long and sometimes unpredictable scan times, limited patient cooperation, difficulty with breath-holding, motion artifacts and high heart rates, paired with the concomitant need for high spatial and temporal resolution due to the small size of cardiac structures. Therefore, the application of AI to pediatric CMR is rapidly evolving across multiple complementary domains—including segmentation, image reconstruction, functional quantification and prognostic stratification, aiming to overcome the time constraints, inter-observer variability, and anatomical complexity that characterize this field [39].

Automation of image analysis represents one of the most significant areas of progress. Karimi-Bidhendi et al. [40] proposed a deep learning–based algorithm for chamber segmentation tailored to pediatric CMR in complex CHD. They addressed the challenge of limited pediatric datasets by incorporating a generative adversarial network (GAN) for synthetic data augmentation. Subsequently, they trained and validated a model that effectively segmented the cardiac chambers, achieving high concordance with expert annotations. Yao et al. [41] applied automated segmentation to a challenging setting of single-ventricle physiology, using data from the multicenter FORCE CMR registry. Despite the heterogeneous anatomy of Fontan patients, the pipeline provided ventricular volume assessment together with data about ventricular mass, stroke volume and ejection fraction, achieving satisfactory agreement with manual analysis.

On the technical side, AI is also transforming image acquisition and reconstruction. Phair et al. [42] introduced an innovative motion-corrected deep learning reconstruction method for three-dimensional whole-heart CMR in patients with CHD. Conventional 3D whole-heart CMR acquisition is often limited by long and unpredictable scan times, as it relies on ECG triggering, respiratory gating and motion-correction strategies, while advanced reconstruction techniques provide high image quality but usually require longer post-processing. To overcome these limitations, the authors adapted a motion-corrected model-based deep learning approach, termed MoCo-MoDL, to reconstruct 3D whole-heart images from highly undersampled free-breathing ECG-triggered acquisitions. In a cohort of 47 adult patients with congenital heart disease, this framework combined non-rigid respiratory motion correction with deep learning–based image reconstruction, achieving diagnostic-quality images with scan times of approximately 2 min and reconstruction times of about 30 s, corresponding to an approximately 240-fold acceleration compared with conventional NR-PROST reconstruction, while maintaining image quality comparable to reference images. In a complementary approach, Montalt-Tordera et al. [43] developed a deep learning-based approach to enhance low-dose contrast-enhanced MR angiography (MRA) in patients with CHD. They used a 3D U-Net to transform low-dose MRA images (acquired with only 20% of the standard gadolinium dose) into enhanced low-dose MRA images designed to approximate standard high-dose MRA. Although unprocessed low-dose images showed significantly poorer image quality, AI-enhanced low-dose images achieved substantially improved signal-to-noise ratio, contrast-to-noise ratio, edge sharpness, and perceived contrast, reaching values comparable to high-dose MRA for most objective and diagnostic measures. Overall image quality and diagnostic confidence were markedly improved, supporting the potential role of AI-assisted contrast dose reduction in patients requiring repeated cardiovascular imaging.

Moving beyond conventional assessment, Govil et al. [44] provided an illustrative example of how AI can integrate multiple analytical steps into a fully automated pipeline for three-dimensional cardiac shape modeling. In patients with repaired tetralogy of Fallot, their approach combined view classification, slice and phase selection, anatomical landmark localization, and myocardial segmentation to generate patient-specific biventricular 3D models, achieving close agreement with manual models while markedly reducing processing time. This study, therefore, represents a broader conceptual shift from isolated AI-assisted image analysis toward integrated computational modeling frameworks capable of capturing the complex anatomy of CHD and supporting future atlas-based, patient-specific clinical applications.

Beyond structural analysis, AI facilitates the extraction of advanced imaging biomarkers and can provide deep pathophysiological insights by identifying disease-specific patterns and supporting prognosis prediction. In this context, Crabb et al. [45] applied a deep learning–based synthetic strain algorithm to cine CMR data from a large multicenter cohort of patients with repaired tetralogy of Fallot. Given the recognized tendency of progressive right ventricular dysfunction in this population and the possible subsequent deterioration of left ventricular mechanics, the study aimed to characterize distinct patterns of left ventricular dysfunction and to explore their relationship with clinical trajectory. Among the four identified phenotypes, the cluster characterized by paradoxical septal motion and reduced septal strain was associated with concomitant right ventricular dysfunction and showed a tendency toward progression to pulmonary valve replacement, suggesting that AI-derived strain analysis may reveal subtle ventricular–ventricular interaction and clinically relevant remodeling patterns not fully captured by conventional volumetric parameters. In line with these findings, Diller et al. [46] showed that deep learning–derived CMR parameters can be integrated into prognostic models, with variables such as right atrial area and right ventricular longitudinal function independently predicting adverse outcomes in patients with repaired tetralogy of Fallot.

Overall, these studies indicate that AI in pediatric CMR is evolving into an integrated framework that encompasses image acquisition, automated analysis, and advanced phenotyping, with the potential to transform the clinical management of congenital heart disease in terms of efficiency, accuracy, and personalization.

### 6.3. Cardiac Computed Tomography

Cardiac computed tomography angiography (CCTA) is another area where AI algorithms are increasingly used. The application of AI is progressively reshaping multiple stages of the imaging workflow, with the primary objective of preserving and eventually improving image quality while minimizing radiation and contrast exposure, which is particularly relevant in children with CHD who often require repeated imaging. In addition, AI supports the visualization and anatomical assessment of complex CHDs, modeling for pre-procedural planning [47].

An important contribution of AI to pediatric CCTA lies in the optimization of acquisition and reconstruction protocols. Zhou et al. [48] demonstrated that the combination of contrast enhancement boost with super-resolution deep learning reconstruction (SR-DLR) enabled an ultra-low-contrast pediatric CHD CTA protocol, achieving a 62.3% reduction in contrast agent dose while maintaining comparable diagnostic image quality. This approach suggests that AI-based reconstruction may support safer imaging protocols without compromising the anatomical information required for clinical decision-making and procedural planning. Beyond contrast reduction, SR-DLR may also improve the intrinsic diagnostic performance of pediatric CCTA by enhancing spatial resolution and reducing image noise. In a subsequent study, Zhou et al. [49] showed that SR-DLR improved visualization of intracardiac structures in pediatric CHD CTA, with potential value for the detection of small anatomical defects, including septal abnormalities, which are particularly relevant when CT is used for detailed preoperative or pre-interventional anatomical assessment.

Parallel efforts have focused on radiation-dose reduction through deep learning–based reconstruction strategies. Yoshiura et al. [50] evaluated infant CCTA using a 70-kVp protocol combined with deep learning reconstruction on 256-detector CT and reported preservation of vascular enhancement and contrast-to-noise ratio despite a 49% reduction in contrast medium and a 41% reduction in radiation dose compared with a conventional 64-MDCT protocol. Similarly, Gulizia et al. [51], in a phantom-based gated cardiac CT study in infants, showed that high-strength deep learning image reconstruction could allow dose reductions up to 64% while preserving image quality.

Beyond technical aspects, AI has been used as a tool to automate segmentation and quantitative evaluation of CT images. The process of automated segmentation is clinically relevant because manual analysis remains time-consuming and operator-dependent, particularly in small children and in patients with complex intracardiac morphology. Yoshida et al. [52] proposed the use of a U-Net–based deep learning model for automated heart segmentation in pediatric cardiac CT, showing that whole-heart and cardiac cavity regions could be accurately extracted from CT datasets. Yao et al. [53] proposed a framework combining deep neural networks and graph matching for whole-heart and great-vessel segmentation in complex CHD. By integrating chamber segmentation with anatomical modelling of the great vessels, this approach addresses one of the major challenges of pediatric CCTA: the need to accurately represent highly variable cardiovascular anatomy, including abnormal ventriculo-arterial connections, vascular malpositions, and complex spatial relationships between intracardiac and extracardiac structures.

The relevance of these approaches lies in their potential to support three-dimensional visualization and 3D printing, technologies that are increasingly used to guide surgical and transcatheter planning in pediatric and adult CHD. Kappanayil et al. [54] have shown that patient-specific 3D-printed cardiac prototypes can improve anatomical understanding, assist surgical decision-making and support the safe execution of complex congenital heart surgery. In this setting, AI should therefore be interpreted as an enabling technology: by accelerating segmentation, reducing observer dependence and improving reproducibility, it may make patient-specific modelling more scalable and clinically accessible.

AI-based CT analysis may also contribute to procedural decisions beyond anatomical visualization alone. In this regard, Szugye et al. [55] developed a deep learning model for automated quantification of total cardiac volume (TCV) from CT scans, achieving high accuracy. TCV-based size matching is a novel technique to compare donor and recipient heart size in pediatric heart transplantation; however, its use is limited by the need for manual segmentation and specialized software and training. The model provided an accurate automatic measurement of TCV from CT images. This approach may facilitate more reproducible preoperative assessment, potentially improving donor–recipient assessment and supporting greater utilization of available grafts.

Overall, these technical and modelling advances collectively illustrate a shift toward a more integrated and technology-driven paradigm in pediatric CCTA, where AI supports not only image reconstruction but also dose optimization and automated analysis, ultimately contributing to safer, more accurate, and scalable evaluation of complex congenital heart disease.

A summary table of the main imaging studies has been included in the Supplementary Materials (Table S1).

## 7. Virtual Reality

Virtual reality (VR) and artificial intelligence (AI) are distinct but increasingly interconnected digital technologies. While VR primarily provides immersive and interactive visualization environments, AI-based algorithms support three-dimensional reconstruction, anatomical modeling and patient-specific simulation, thereby facilitating the generation of advanced virtual environments from multimodality imaging datasets. Cardiovascular medicine represents a particularly fertile field for the convergence of AI and VR, as increasingly sophisticated platforms are being developed for medical education, procedural training, surgical planning and precision medicine applications.

Simulation is now a well-established teaching method capable of promoting learning and training through controlled, safe, and reproducible educational experiences. It has been widely demonstrated that learning is more effective when learners have greater control over the content and can actively interact with the concepts presented. Alongside traditional high-fidelity simulation techniques, such as those based on the use of manikins, virtual reality is gradually gaining ground; it constitutes a valid alternative and is becoming increasingly widespread in the medical and healthcare fields. In pediatric cardiology—a field that requires a deep understanding of highly complex three-dimensional structures—VR is emerging as an innovative and promising tool for both healthcare professionals and patients. Starting from two-dimensional images obtained through conventional imaging methods and appropriately integrated, VR enables the reconstruction of immersive, explorable, and interactive three-dimensional models. These models are capable of detecting the user’s position and movements, adapting the visualization in real time and providing visual, auditory, or tactile feedback. For example, tilting the head or rotating the torso results in an immediate change in the perspective of the virtual environment. This multisensory feedback generates a genuine sense of immersion, allowing for free and intuitive exploration of the represented space.

Therefore, the first and most immediate area of application for virtual reality is training and education [56]. In pediatric cardiology, the complete immersion in the subject matter made possible by VR translates into the ability to virtually observe and “dissect” hearts affected by congenital heart defects, even simulating surgical correction procedures. A prime example is the Stanford Virtual Heart Project [57], an innovative initiative resulting from a collaboration between Stanford’s Lucile Packard Children’s Hospital and Silicon Valley technology companies. The project uses virtual reality to enhance physician training and has demonstrated a significant improvement in learning outcomes.

The potential of virtual reality, however, is not limited to the educational field. In recent years, there has been growing interest in its use for diagnosis and surgical planning of cardiac conditions in both pediatric and adult patients [58,59,60,61]. In this context, Pushparajah et al. [62] demonstrated the feasibility of integrating three-dimensional echocardiographic datasets into immersive VR systems for planning surgical repair of atrioventricular valves in patients with congenital heart disease. Mejia et al. [63] proposed AI-driven reconstruction approaches capable of generating patient-specific virtual models of complex congenital cardiac defects, further supporting the integration between AI-based image processing and immersive visualization technologies. Additionally, Ghosh et al. [59] described the implementation of a clinical three-dimensional modeling program integrating advanced imaging, segmentation, and immersive visualization technologies to guide pediatric cardiothoracic surgery and catheter-based interventions in complex CHD anatomies.

Potential practical applications include assessing donor–recipient matching in heart transplantation and even simulating post-surgical hemodynamic flows, with the potential to predict the outcomes of different surgical approaches [61].

In conclusion, virtual reality represents a highly promising technology for pediatric cardiology, particularly suited to a discipline based on complex spatial relationships. Although statistical evidence on clinical outcomes is still limited, VR is set to play an important role in training, surgical planning, and patient preparation.

## 8. Limitations

Despite the rapid expansion of artificial intelligence in healthcare, its application in clinical medicine still presents several significant limitations.

First, from a technical and data-related perspective, AI systems require large and high-quality datasets in order to achieve reliable performance. This requirement is particularly challenging in pediatric cardiology, since many congenital heart diseases are rare, anatomically heterogeneous, and characterized by wide variability in age, body size, physiology, imaging protocols, clinical presentation and surgical corrections. As a result, datasets are often small, fragmented or single-center, which may limit model reproducibility and generalizability across different institutions and patient populations. In addition, differences in data acquisition, image quality, annotation standards, and outcome definitions may further impair the development of robust and clinically transferable AI models.

Organizational barriers also represent an important limitation to clinical implementation. AI tools must be integrated into pre-existing clinical workflows, electronic health records and platforms and multidisciplinary decision-making processes. AI systems may subsequently increase workload or remain confined to research settings rather than being adopted in routine care.

Social and cultural factors may also influence implementation, as clinicians, patients, and families may be reluctant to trust AI-supported recommendations, particularly when the output is difficult to interpret or when the role of the algorithm in clinical decision-making is unclear. These aspects are especially relevant in pediatric care, where communication with parents and caregivers is a central component of clinical management.

A further major limitation is represented by the limited explainability and transparency of many AI systems. From a methodological and technical perspective, this issue corresponds to the so-called “black-box problem”. The term refers to the fact that many AI models, particularly deep learning algorithms, can generate accurate outputs without providing a clear explanation on how the final conclusion was reached (i.e., which variables most strongly influenced the prediction, what limitations or potential biases may have affected the algorithmic output). In other words, the internal reasoning of the model often remains inaccessible or difficult to interpret, even for developers and clinicians. Among other limitations, we should remind the regulatory compliance and device classification problems, the regulatory approval timeline, and the algorithm degradation over time.

Examples can illustrate why limited explainability represents a concrete obstacle to clinical implementation. In cardiovascular imaging, convolutional neural networks may correctly classify images or clips from various imaging modalities, but without explanation, it may be unclear whether the algorithm based its prediction on relevant anatomical or functional features (i.e., ventricular morphology and size, valve anatomy, Doppler patterns, myocardial tissue characteristics). The same issue applies to AI-enabled electrocardiography. Deep learning algorithms have shown the ability to detect or predict conditions such as left ventricular dysfunction, atrial fibrillation, hypertrophic cardiomyopathy, pulmonary hypertension, or other structural heart diseases from ECG patterns that may appear subtle or even normal to human interpretation. Although this ability is promising, the lack of interpretability may limit clinical confidence if the model does not indicate which ECG features contributed to the prediction (i.e., QRS morphology, repolarization abnormalities, conduction delay, voltage criteria, rhythm variability, or interval changes). In pediatric cardiology, these limitations are even more relevant: heart diseases present with marked anatomical variability; ECG interpretation is strongly influenced by age, body size, heart rate, and developmental changes.

Limited explainability may also conceal hidden algorithmic bias. For instance, an AI model trained predominantly on images or ECGs from specific age or ethnic groups or disease subtypes may perform less accurately in underrepresented populations. This problem may be amplified in pediatric cardiology, given the rarity of individual diagnoses and the imbalance between common and complex lesions. Without explainability and subgroup performance assessment, these biases may remain undetected and could contribute to diagnostic errors or healthcare disparities.

Ethical and regulatory aspects, including privacy, accountability, equity and medical liability, are briefly discussed in the dedicated section. However, these issues remain the subject of ongoing debate and research and fall beyond the primary scope of the present review.

These limitations are amplified in pediatric cardiology and congenital heart disease, where patient populations are vulnerable, clinical decisions are often complex and individualized, and diagnostic or therapeutic strategies must be clearly explained within multidisciplinary teams and to families. Therefore, before AI can be safely and effectively integrated into routine pediatric cardiovascular care, future research must address not only algorithmic performance but also data quality, external validation, workflow integration, interpretability, clinician acceptance, and real-world clinical impact.

## 9. Ethics and Regulation

The growing use of artificial intelligence in medicine, particularly pediatric cardiology, raises relevant ethical and regulatory challenges. Its application introduces concerns related to transparency, informed consent, data protection and privacy, data quality and bias, accountability [64], medical liability [65,66], and the trust of patients and healthcare professionals. These matters are of particular relevance in pediatric and congenital heart disease, where model generalizability is limited by small datasets. Additionally, considering that most patients are minors, ethical implications and regulatory considerations become even more critical.

One of the major challenges limiting the integration of AI algorithms into clinical practice is the explainability of many AI systems (“black-box problem”). Reduced transparency could hinder informed consent, increase the risk of automation bias (mainly a clinical overreliance on the AI results and consequently deskilling of clinicians), and complicate accountability when AI-supported decisions contribute to diagnostic or therapeutic management. To address this challenge, patients and families should always be informed when AI tools contribute to diagnosis or management, including the intended role of the algorithm, the methods and type of data used, the limitations, including the possibility of false-positive or false-negative outputs. Current informed consent models are often insufficient, as they rarely explain with accessible language the algorithmic logic, data use, bias, and accountability. Therefore, AI-informed consent should include plain-language explanations, visual aids if appropriate, disclosure of data protection safeguards, and clarification of clinical responsibility [67]. Medical liability for AI-related errors (i.e., false-positive or false-negative responses) should be interpreted through measurable performance and clinical-impact metrics rather than through global accuracy alone [68]. Liability assessment should consider sensitivity, specificity, positive and negative predictive value, calibration, and area under the receiver operating characteristic curve, together with clinical context and disease prevalence. For segmentation tasks, metrics such as Dice Similarity Coefficient or Intersection over Union may quantify technical accuracy, but they do not automatically establish clinical safety unless linked to clinically meaningful endpoints. From a medico-legal perspective, responsibility should depend on where the failure occurred. If the algorithm was inadequately trained, poorly validated, biased, or insufficiently monitored, liability may involve developers, manufacturers, or institutions. If the tool was used outside its approved indication, accepted without critical review, or not integrated into an appropriate clinical workflow, responsibility may involve the healthcare provider or institution. Importantly, even highly performing AI systems can produce errors; therefore, clinicians should be trained to interpret AI outputs probabilistically rather than deterministically. For this reason, AI outputs should be documented as supportive evidence, not as autonomous decisions.

Last but not least, the problem of equity in healthcare represents a further concern. AI tools are currently being developed mainly in well-resourced institutions, and their uneven availability may further deepen existing healthcare disparities. Ensuring equitable access to AI-based technologies, for example, through open-access platforms, will therefore represent an important challenge for the future.

These ethical concerns are increasingly reflected in the evolving regulatory framework for AI in healthcare. In Italy, as in other European Union Member States, the normative framework is provided by the European Union and the European Commission on Artificial Intelligence. On its website page, AI is presented as a technology with considerable potential to improve healthcare delivery; however, its use must take place within clear regulatory frameworks, particularly when it is used for clinical decision support in patient care. Further details are provided in Supplementary Text S1 in the Supplementary Materials.

## 10. Future Directions

The future development of AI in pediatric cardiology should move beyond the mere improvement of diagnostic or prognostic accuracy and focus on the creation of explainable and reliable systems.

Future efforts should be aimed at improving the diagnostic and prognostic capacities of AI tools. However, considering that blind reliance on algorithmic outputs is especially inappropriate in pediatric cardiology, bridging the gap between algorithmic performance and real-world clinical implementation is the real challenge. In this context, explainable AI represents one of the most important future directions. Current approaches include saliency maps, gradient-weighed class activation mapping (Grad-CAM), attention maps, feature-importance ranking, interpretable machine learning models, uncertainty estimation, and confidence scoring systems [69].

These methods aim to make AI outputs more transparent by showing which data elements most influenced the prediction and whether the model’s reasoning is clinically plausible. In cardiovascular imaging, for example, explainable AI systems should be able to indicate whether a prediction was driven by clinically meaningful anatomical or functional features, such as ventricular morphology, valve anatomy, septal defects, outflow tract abnormalities, great vessel relationships, chamber volumes, myocardial mass, scar burden, or flow parameters. In echocardiography, CMR, and CCT, visualization-based XAI approaches (i.e., saliency maps) may help clinicians verify whether the algorithm focused on relevant cardiac structures rather than on non-clinical confounders such as image quality, acquisition protocol, scanner type, annotations, or background artifacts. Similarly, in AI-enabled electrocardiography, explainability tools may help identify which ECG features, waveforms, intervals, or repolarization patterns contributed to the prediction.

Future research should also focus on combining explainability with uncertainty estimation and confidence scoring. In clinical practice, it is not sufficient for an AI model to provide a prediction; clinicians also need to know how reliable that prediction is and in which situations the model may be uncertain or potentially unreliable. This is especially important in rare or complex congenital heart diseases, where the algorithm may encounter phenotypes that were underrepresented or absent in the training dataset. By providing interpretable outputs together with measures of confidence, AI systems could better support clinical reasoning, reduce the risk of automation bias, and help physicians decide when AI recommendations should be accepted, questioned, or disregarded.

Another important future direction will be the development of XAI systems that are designed around human–AI collaboration. Rather than replacing expert judgment, AI should function as a transparent decision-support tool integrated into multidisciplinary workflows. In pediatric cardiology, explainable AI could support image acquisition, automated quantification, lesion recognition, risk stratification, procedural planning, longitudinal follow-up, and shared decision-making, while leaving final clinical responsibility to trained physicians. To achieve this goal, future studies should evaluate not only the technical performance of AI models but also the clarity and usefulness of their explanations, their impact on clinician confidence and decision-making, their integration into clinical workflow, and their ability to improve patient outcomes.

Ultimately, improving explainability may enhance clinician trust, facilitate informed consent, support shared decision-making with patients and families, clarify accountability, reduce the risk of hidden bias and automation bias, and promote safer integration of AI into routine pediatric cardiovascular care. Therefore, explainability should not be considered an optional feature, but a fundamental requirement for the responsible translation of AI from research to clinical practice.

## 11. Conclusions

AI is progressively reshaping pediatric cardiology, bridging current clinical practice with future precision medicine. A major strength of AI lies in its ability to process large volumes of heterogeneous data and identify complex, non-linear patterns, often outperforming traditional statistical approaches. As highlighted in this commentary, AI-driven approaches have already demonstrated significant value across multiple domains, including electrocardiographic analysis, digital auscultation, and advanced multimodality imaging. These technologies enable more accurate, reproducible and efficient diagnostic pathways, particularly in complex conditions such as congenital heart disease, while also supporting telemedicine and expanding access to specialized care. Beyond current applications, the integration of AI with emerging technologies—such as virtual reality and patient-specific digital models—opens new perspectives in procedural planning and personalized treatment strategies.

However, much of the current evidence is derived from retrospective, single-center studies or highly selected datasets, raising concerns about overfitting and limited generalizability. This issue is particularly relevant in pediatric cardiology, where smaller patient populations and the heterogeneity of congenital heart disease (CHD) significantly constrain the development of robust models.

The transition from promising innovation to routine clinical implementation requires overcoming key limitations, including the scarcity of large pediatric datasets, challenges in generalizability and the need for transparent and explainable models. Ethical, legal and organizational aspects must also be carefully addressed.

Looking forward, the future of pediatric cardiology will likely be characterized by a synergistic interaction between clinicians and intelligent systems. In this evolving landscape, AI is not intended to replace the physician but to enhance clinical reasoning and decision-making. Its responsible and validated integration into healthcare systems has the potential to significantly improve outcomes and quality of life for children with cardiovascular disease.

## Data Availability

No new data were created or analyzed in this study.

## Supplementary Materials

The following supporting information can be downloaded at: https://www.mdpi.com/article/10.3390/pediatric18030070/s1. Supplementary Text S1: Overview of the European regulatory framework for artificial intelligence in healthcare; Table S1: Summary of artificial intelligence applications in cardiovascular pediatric imaging.

## Abbreviations

The following abbreviations are used in this manuscript:

AI	Artificial Intelligence
ML	Machine Learning
DL	Deep Learning
CHD	Congenital Heart Disease
CNN	Convolutional Neural Networks
RNN	Recurrent Neural Networks
ECG	Electrocardiogram
CMR	Cardiac Magnetic Resonance
CCT	Cardiac Computed Tomography
VR	Virtual Reality
