# Supplementary material for: Artificial Intelligence in Pediatric Cardiology: Present Applications and Future Directions
Source: Pediatr Rep. 2026 May 25;18(3):70. doi: 10.3390/pediatric18030070 (PMC13306153; doi:10.3390/pediatric18030070)
Supplement: Supplementary file 1 [file pediatrrep-18-00070-s001.zip › pediatrrep-4301755-supplementary.pdf]

Supplementary Text S1: Overview of the European regulatory framework for artificial intelligence in healthcare

The main European regulatory reference is the Artificial Intelligence Act, which entered into force on August 1st, 2024 and aims to promote responsible development and deployment of AI across the European Union [1]. The document emphasizes that high-risk AI systems, including most AI-based software intended for medical purposes, must comply with specific requirements. In particular, the Commission refers to risk-mitigation systems, high-quality datasets, clear information for users, and human surveillance. The European AI Office has also been established as the European centre of expertise for AI, with a key role in supporting the implementation of the AI Act. More broadly, for general-purpose AI models, the final Code of Practice for providers of General-Purpose AI models [2], published in July 2025, provides additional guidance for general-purpose AI systems.

The European Commission also addresses some of the core ethical and practical issues raised by AI in medicine, particularly data governance and liability. An important component of the European framework is the European Health Data Space (EHDS) [3], which entered into force in 2025 and provides specific rules for the health sector, taking into account the particular sensitivity of health data. It builds on broader European legal frameworks, including the General Data Protection Regulation (GDPR) [4], the Data Governance Act, the Data Act, and the Network and Information Systems Directive. The Commission explains that the implementation of AI in medicine requires access to diverse, large-scale and high-quality health data in order to ensure accuracy, robustness, and fairness across different populations. The EHDS aims to facilitate and, at the same time, regulate both the primary and secondary use of electronic health data, where primary use refers to direct patient care and secondary use refers to research, innovation, policy-making, and regulatory activities.

The page also discusses the new Product Liability Directive (PLD) [5], which updates European rules on liability for defective products. A key point is that software, including AI systems, is considered a product for liability purposes. This means that, if defective AI software causes harm, the manufacturer may be held liable for compensation. According to the directive of the Commission, software such as AI systems fall within the scope of strict liability, regardless of how they are supplied or used. In addition, software developers or producers, including providers of AI systems under the AI Act, must be treated as manufacturers. This means that for AI- based medical tools, responsibility for harm may involve clinicians, healthcare institutions and developers, manufacturers and providers of AI systems, especially when defects relate to inadequate validation, monitoring or post-market performance.

This regulatory landscape is complemented by initiatives aimed at facilitating safe clinical implementation. One of these is represented by AICare@EU, an initiative proposed by the Directorate-General for Health and Food Safety. Despite progress in the research and development of AI/ML-based medical devices, their availability on the market and integration into routine clinical practice remain limited. AICare@EU therefore aims to address the barriers to clinical adoption of AI, including technological and data-related, legal and regulatory, organizational and economic, as well as social and cultural barriers. Among the integrated efforts proposed, priority is also given to the prevention of cardiovascular diseases.

Other international authorities, including the World Health Organization (WHO), the U.S. Food and Drug Administration (FDA) and the International Medical Device Regulators Forum (IMDRF) have also issued guidance on this topic [6-8]. A detailed discussion of these frameworks goes beyond the scope of this review, but these documents equally offer important observations in this regard.

References

1. European Parliament and Council of the European Union. Regulation (EU) 2024/1689 of 13 June 2024 laying down harmonised rules on artificial intelligence (Artificial Intelligence Act). Official Journal of the European Union. 2024;L 2024/1689
2. European Commission. The General-Purpose AI Code of Practice. Shaping Europe's Digital Future. Published July 2025
3. European Parliament and Council of the European Union. Regulation (EU) 2025/327 of 11 February 2025 on the European Health Data Space. Official Journal of the European Union. 2025;L 2025/327.
4. European Parliament and Council of the European Union. Regulation (EU) 2016/679 of 27 April 2016 on the protection of natural persons with regard to the processing of personal data and on the free movement of such data — General Data Protection Regulation. Official Journal of the European Union. 2016;L119:1–88.
5. European Parliament and Council of the European Union. Directive (EU) 2024/2853 of 23 October 2024 on liability for defective products and repealing Council Directive 85/374/EEC. Official Journal of the European Union. 2024;L 2024/2853.
6. World Health Organization. Ethics and governance of artificial intelligence for health: guidance on large multi-modal models. Geneva: World Health Organization; 2025.
7. U.S. Food and Drug Administration. Artificial Intelligence/Machine Learning (AI/ML)-Based Software as a Medical Device (SaMD) Action Plan. FDA; 2021
8. U.S. Food and Drug Administration. Good Machine Learning Practice for Medical Device Development: Guiding Principles. FDA; updated 2025.

Supplementary Table S1: Summary of artificial intelligence applications in cardiovascular pediatric imaging.

Field	Study	Year	Population / dataset	Input data	AI method	Training set	Testing / validation set	Main findings	Limitations
Echocardiography	Gearhart et al.	2022	642 pediatric echocardiograms (patients aged 0–19 years); 27,948 clips.	Echocardiographic images including anatomic sweeps, color Doppler and Doppler tracings.	CNN for automated classification of 27 standard pediatric echocardiographic views.	12,067 images.	6,197 validation images.	Overall view-classification accuracy 90.3%, similar across age groups	Single-center study; exclusion of some severe/rare CHD forms; model focus on view classification rather than diagnosis.
Echocardiography	Jiang et al.	2023	1376 children and their echocardiographic views; 14,838 images.	Echocardiographic videos using seven standard views.	CNN for detection of pediatric CHD.	Splitting of the internal dataset in 8:1:1 (training/validation/testing).	Splitting of the internal dataset in 8:1:1 (training/validation/testing).	AUC up to 0.91 and accuracy up to 92.3% when appropriate image modalities were used; stable performance under shear-transformation interference.	Single-center dataset; performance depended on appropriate input image modality; no external validation reported.
Echocardiography	Erno et al.	2023	66 preterm infants; 2,527 echocardiographic video clips.	Echocardiography video clips including color Doppler PDA clips.	CNN with attention-based frame aggregation for clip-level PDA classification.	1,145 color Doppler clips, labeled as PDA-positive and PDA-negative.	Internal 6-fold cross-validation with patient-level split: in each fold, 44 patients for training, 11 for validation, and 11 for testing; no external validation.	Strong clip-level and study level-sensitivity	Further model development and external validation required; focus on PDA presence rather than full hemodynamic assessment.
Echocardiography + clinical data	Sharma et al.	2025	174 preterm infants treated pharmacologically for PDA; 1,926 echocardiographic clips.	Pretreatment echocardiograms alone or echocardiograms plus perinatal clinical data.	Multimodal CNN for prediction of pharmacologic PDA closure; compared with imaging-only CNN, logistic regression and random forest.	121 infants, 1,387 clips.	53 infants, 539 clips.	Multimodal CNN AUC 0.82, outperforming imaging-only CNN AUC 0.66, logistic regression AUC 0.66 and random forest AUC 0.74.	Internal validation only; neonatal echo quality variability; limited interpretability despite saliency-map analysis.
Echocardiography	Diller et al.	2019	132 adults with systemic RV (92 TGA after atrial switch, 40 ccTGA); 67 normal controls.	Routine transthoracic echocardiograms frames dorm apical four-chamber and parasternal short-axis views.	CNN for diagnosis classification; U-Net for systemic ventricular segmentation.	>100,000 frames; segmentation model trained on manually annotated frames with 80% training/validation split.	20% test set from patients not used for training.	Overall diagnostic accuracy 98%; systemic ventricle correctly identified in all individuals; Dice 0.79–0.88 depending on diagnosis.	Adult CHD population rather than pediatric-only; external validation required.
Echocardiography	Sun et al.	2025	Multicentre retrospective cohort of 29,142 children with perimembranous ventricular septal defect from six tertiary centres in China.	Electronic echocardiographic reports and hospital information system medical records.	NLP-based semantic extraction of structured variables from raw reports; LASSO for predictor selection; comparison of CoxPH, glmnet, random survival forest and DeepSurv models.	Dataset 1: 27,269 patients randomly split into 70% training (and 30% validation).	Internal validation: 30% of Dataset 1. Independent testing dataset: 1,873 patients from two centres.	Random survival forest showed the best performance. The model predicted spontaneous closure at 1, 3 and 5 years with AUCs of 0.95, 0.97 and 0.98 in the testing set, respectively. Eleven NLP-derived predictors were selected, mainly related to defect morphology and patient age.	Retrospective design; data from tertiary referral centres may introduce selection bias; further external testing and prospective clinical validation needed; use of report-derived information rather than raw echocardiographic images.
Echocardiography	Guo et al.	2021	127 2D paediatric echocardiography from healthy children aged 0–10 years; 4,485 extracted images.	2D echocardiographic four-chamber views.	Dual attention enhancement feature fusion CNN for LV, LA and apical-triangle segmentation and quantitative parameter extraction.	100 videos, 3,654 images.	Internal testing: 27 videos / 831 images; no external validation.	Proposed model achieved good segmentation performance and enabled automatic extraction of clinical parameters for diagnosis and planning.	Technical segmentation study; relatively limited dataset; clinical outcome impact not directly assessed.
Echocardiography	Meza et al.	2018	Multicentre cohort of 651 neonates with critical left heart obstruction.	136 qualitative and quantitative variables from baseline pre-intervention transthoracic echocardiograms;	Unsupervised hierarchical agglomerative cluster analysis.	Not applicable; full cohor used for unsupervised clustering.	No separate training/testing split or external validation set.	Three clusters emerged: 215, 338 and 98 patients; groups differed by aortic valve atresia, LV size, intervention strategy and mortality.	Not a supervised diagnostic AI model; no external validation; dependent on baseline echo-variable availability and core-lab measurement.
Echocardiography	Lukyanenko et al.	2026	217,435 internal echos + 3,096 external referral echos.	Full echocardiographic studies.	EchoFocus-Measure: multi-task deep learning model with PanEcho backbone and transformer attention.	80% internal dataset	20% internal testing set plus external referral studies.	Automatically extracted 18 quantitative and 10 qualitative assessments; LVEF MAE 2.8% internally and 3.8% externally; qualitative AUROC 0.88–0.95 internally and 0.73–0.86 externally.	Preprint not yet peer-reviewed; qualitative performance lower in external testing; dependent on report-derived labels.

CMR	Karimi-Bidhendi et al.	2020	64 pediatric subjects (2-18 years) with complex CHD (including ToF, DORV, TGA, cardiomyopathy, coronary artery anomaly, pulmonary stenosis/atresia, truncus arteriosus and aortic arch anomaly).	Short-axis cine CMR images at end-diastole and end-systole; LV and RV segmentation.	Deep FCN with GAN-based synthetic data augmentation.	26 patients dataset, augmented with synthetic CMR images and corresponding chamber masks.	Internal test set of 38 patients; additional testing with ACDC dataset.	Dice: LV 91.0% (ED) and 86.8% (ES); RV 84.7% (ED) and 80.6% (ES), outperforming U-Net and cvi42.	Small pediatric CHD dataset; heterogeneous anatomy; no large multicenter external validation.
CMR	Diller et al.	2020	372 patients with repaired ToF from a nationwide prospective study; median follow-up 10 years.	CMR cine frames in short-axis and long-axis views; combined with clinical and ECG data.	DL-based automated image analysis for cardiac dimensions/function, followed by prognostic modeling.	Local CMR dataset form 42 patients; 836 short-axis image/mask pairs and 1045 long-axis image/mask pairs used for U-Net training.	External multicenter national ToF cohort from 14 German institutions.	DL-derived right atrial area and RV long-axis strain predicted death, aborted cardiac arrest or ventricular tachycardia; composite score identified higher-risk patients.	Proof-of-principle using 2D data; did not use full 3D volumetric DL analysis; larger training sets and transfer learning may be required.
CMR	Govil et al.	2023	Multicentre cohort of 123 repaired ToF patients (internal and external centres)	CMR images for automated generation of 3D biventricular shape models.	End-to-end DL pipeline for view classification, slice/phase selection, landmark localization and myocardial segmentation.	111 rTOF patients from internal sites	Internal validation set: 12 rTOF patients from the same internal sites; independent test set: 30 rTOF patients, including 18 external cases.	Automated shape models achieved MAE 1.9 +/- 0.5 mm at ED and 2.1 +/- 0.7 mm at ES, similar to image voxel resolution; global ventricular measures agreed with manual models.	Relatively small test cohorts; pipeline focused on repaired ToF; broader CHD validation needed.
CMR	Yao et al.	2023	Multicenter FORCE CMR registry, 250 CMR examinations in patients with Fontan/single-ventricle physiology.	Short-axis cine cardiac CMR images	Three-model DL pipeline: CNN classifier for short-axis cine stacks, U-Net 3+ cropping and U-Net 3+ ventricular segmentation.	175 CMR exams	Internal validation set: 25 examinations. Test set: 50 examinations. Additional testing on 475 unseen examinations.	Dice scores: EDV 0.91 and ESV 0.86; high ICCs for EDV/ESV (>0.97); satisfactory segmentation in 68% of unseen cases and minor adjustment in 26%.	Some cases still required manual correction; ventricular mass agreement was more moderate; registry heterogeneity remains challenging.
CMR / MRA	Montalt-Tordera et al.	2021	CHD patients undergoing contrast-enhanced MR angiography (1157 retrospective and 40 prospective patients).	3D Contrast-enhanced cardiovascular MR angiography images.	Neural network enhancement of low-dose 3D MRA to reduce gadolinium contrast dose.	1056 retrospective synthetic low-dose/high-dose MRA pairs.	Internal validation: 117 synthetic paired volumes. Prospective test set: 40 patients with true low-dose and high-dose MRA acquisitions.	Enhanced low-dose MRA improved SNR, CNR, edge sharpness, perceptual contrast and diagnostic confidence; sensitivity/specificity improved from 0.824/0.921 to 0.882/0.960.	Perceptual sharpness remained lower than high-dose MRA; prospective test cohort was limited.
CMR	Phair et al.	2024	47 adult CHD patient undergoing 3D whole-heart CMR.<br><br>datasets used for training; 8 CHD patient datasets for evaluation.	Free-breathing ECG-triggered 3D whole-heart CMR data.	Motion-corrected model-based deep learning reconstruction (MoCo-MoDL) with non-rigid motion estimation and denoising U-Net regularization.	39 CHD datasets.	8 CHD test datasets with prospective seven-fold undersampling; comparison with NR-PROST and reference images.	Seven-fold undersampled scans took 2.1 +/- 0.3 min; reconstruction was ~30 s, ~240 times faster than NR-PROST, with comparable or better image quality.	Small evaluation cohort; adult CHD population; focused on 3D whole-heart acquisition rather than pediatric-only CMR.
CMR	Crabb et al.	2025	Multicenter population of 198 patients with repaired ToF and 21 healthy controls.	Short-axis cine SSFP CMR images.	Deep learning synthetic strain (DLSS) to measure regional LV strain and dyssynchrony; clustering analysis of contraction patterns; Cox regression for progression to PVR.	Not applicable: pretrained/previously developed DLSS applied to cohort.	No separate testing/validation split. Comparison with healthy controls; association with CMR metrics and progression to pulmonary valve replacement.	rTOF patients showed decreased septal radial strain, increased lateral wall strain and greater dyssynchrony; clustering identified four LV contraction patterns, including paradoxical septal motion.	Retrospective design; strain patterns require prospective validation and integration with clinical endpoints.
CCTA	Gulizia et al.	2024	Anthropomorphic pediatric phantom simulating a 6-month-old infant, scanned with ECG-gated cardiac CT using a clinical CHD protocol.	ECG-gated cardiac CT acquisitions at four radiation-dose levels.	Deep learning image reconstruction (DLIR) compared with ASIR-V iterative reconstruction.	Not applicable: phantom-based evaluation of a commercially available DLIR reconstruction algorithm.	Phantom testing across repeated acquisitions at different radiation dose levels; DLIR-H compared with ASIR-V50 as the clinical standard.	High-strength DLIR increased detectability by 2.4% and discrimination performance by 20.9% versus ASIR-V 50. Similar detectability could theoretically be preserved with 64% dose reduction.	Phantom rather than patient study; clinical diagnostic accuracy and patient-specific motion effects not assessed.
CCTA	Yoshiura et al.	2026	Single-centre cohort of 30 infants with suspected CHD undergoing cardiac CT angiography. ; 256-MDCT group n=12 and 64-MDCT group n=18.	Cardiac CTA images acquired with 70 kVp 256-MDCT using reduced contrast medium versus 80 kVp 64-MDCT using standard contrast dose.	Deep-learning reconstruction combined with low-tube-voltage wide-detector CT. Compared with ASiR 30% iterative reconstruction on 64-MDCT.	Not applicable; comparative CT protocol study.	Comparative clinical testing: 12 infants in the 256-MDCT/DLR group versus 18 infants in the 64-MDCT/ASiR group. No separate validation cohort.	70 kVp 256-MDCT with DLR maintained vascular enhancement and CNR while reducing contrast medium by 49% and radiation dose by 41% compared with 80 kVp 64-MDCT with ASiR.	Retrospective single-center study; small sample size; protocol and scanner-specific findings.
CCTA	Szugye et al.	2025	314 subjects aged 0-30 years, including normal hearts and heart disease cohorts.	CT images with manually segmented total cardiac volume (TCV).	Custom 3D-CNN using DenseNet architecture combined with residual blocks for automated TCV segmentation.	270 subjects.	44 subjects, including 36 normal hearts and 8 with heart disease.	Average Dice similarity coefficient 0.94 +/- 0.03; mean absolute percent error for TCV estimation 5.5%. Performance was better in normal hearts than in transplant candidates with CM/CHD.	Single-center study; limited number of heart-disease cases; model generalizability and performance in complex CHD require multicenter validation.

CCTA	Zhou et al.	2026	91 pediatric patients aged 1-10 years with suspected CHD undergoing free-breathing CCTA.	CCTA images reconstructed using SR-DLR, conventional DLR and hybrid iterative reconstruction.	Super-resolution deep learning reconstruction (SR-DLR) for image-quality improvement and lesion visualization.	Not applicable: comparative reconstruction algorithm study.	Same cohort reconstructed and evaluated with SR-DLR, C-DLR and HIR. Diagnostic performance was validated against surgery or echocardiography. No separate external validation cohort.	SR-DLR reduced image noise, increased SNR/CNR and improved subjective image quality. Diagnostic accuracy reached 99.12% with sensitivity 99.07%; sensitivity improved especially for ASD/VSD and multi-perforated defects.	Single-center cohort; reconstruction algorithm and scanner-specific; requires broader external validation and outcome-based assessment.
CCTA	Zhou et al.	2025	Single center cohort of 72 pediatric CHD patients, randomized to low-contrast CE-Boost group (n=36) or standard contrast CE-CT group (n=36).	Pediatric CHD CTA images acquired with low contrast dose plus CE-Boost, versus standard contrast dose; both groups reconstructed with SR-DLR.	Contrast-enhancement boost post-processing combined with super-resolution deep learning reconstruction.	Not applicable: prospective comparative protocol study.	Clinical comparison between CE-Boost group, n=36, and standard CE-CT group, n=36. Diagnostic performance was compared with surgical or echocardiographic reference standards.	CE-Boost plus SR-DLR reduced contrast-agent dose by 62.3% while maintaining comparable image quality; CT values, SNR, CNR and subjective scores remained stable.	Single-center study; relatively small sample; focused on contrast-dose reduction rather than long-term clinical outcomes.
CCTA	Yoshida et al.	2022	20 pediatric patients aged <15 years, with suspect of CHD or other abnormalities but without confirmed cardiac anomalies.	Approximately 7,500 contrast-enhanced cardiac CT images with whole-heart and chamber labels.	Two-step U-Net segmentation: first whole-heart segmentation, then separate segmentation of RA, RV, LA and LV.	Leave-one-subject-out approach: 19 patients used for training at each iteration.	One patient held out for evaluation at each iteration; performance averaged across all 20 patients. No external validation cohort.	Mean Dice similarity coefficient was 0.954 ± 0.013 for whole-heart segmentation. Chamber segmentation also showed good performance, with median Dice values of 0.872 for RA, 0.880 for RV, 0.867 for LA, 0.885 for LV and 0.893 for all chambers.	Small single-centre dataset; only patients without major cardiac morphological abnormalities were included; 2D U-Net did not account for body-axis continuity; segmentation accuracy varied in some age groups, partly due to differences in contrast conditions and CT protocols.
CCTA	Yao et al.	2023	68 from patients with CHD, aged 1 month to 21 years; dataset included 14 CHD types, with 37 simple and 31 complex CHD cases.	3D cardiac CTA images; seven manually labelled cardiovascular structures: LV, RV, LA, RA, myocardium, aorta and pulmonary artery.	Combined deep learning and graph-matching framework: 3D U-Net for chamber/myocardial segmentation, 2D U-Net for blood-pool segmentation, and graph matching for classification of the aorta, pulmonary artery and anomalous vessels.	Fourfold cross-validation: 51 images used for training in each fold.	17 images used for testing in each fold; clinical evaluation performed by two cardiovascular imaging specialists using the Van Praagh classification system. No external validation cohort.	The proposed method achieved a mean Dice score of 78.3 ± 10.8%, outperforming Seg-CNN by approximately 12% overall. Dice scores improved for all seven structures, especially the aorta. Clinical evaluation showed correct atrioventricular connections in 70/71 cases, ventriculoarterial connections in 69/71 cases, and great-vessel relationships in 70/71 cases.	Relatively small single-centre dataset; some segmentation errors persisted, especially in complex CHD and anomalous vessel connections; down-sampled 3D images were used because of GPU memory limits; Dice score alone may not fully capture clinically relevant anatomical errors.
